# 3,10,14,21-Tetra­kis(4-meth­oxy­phen­yl)penta­cyclo­[11.8.0.0^2,11^.0^4,9^.0^15,20^]henicosa-1(21),2,4(9),5,7,10,13,15(20),16,18-decaen-12-one chloro­form monosolvate

**DOI:** 10.1107/S1600536814016389

**Published:** 2014-07-23

**Authors:** S. Gopinath, P. Narayanan, K. Sethusankar, Meganathan Nandakumar, Arasambattu K. Mohanakrishnan

**Affiliations:** a Department of Physics, RKM Vivekananda College (Autonomous), Chennai 600 004, India; b Department of Organic Chemistry, University of Madras, Guindy Campus, Chennai 600 025, India

**Keywords:** crystal structure, naphthalene derivatives, hydrogen bonding, hydrogen-bonding chains, polycyclic compounds

## Abstract

The asymmetric unit of the title compound, C_49_H_36_O_6_·CHCl_3_, contains half an organic mol­ecule, the complete mol­ecule being generated by the operation of a crystallographic twofold rotation axis, and half a highly disordered chloro­form mol­ecule. The contribution to the diffraction pattern of the latter was removed using the program SQUEEZE in *PLATON* [Spek (2009 ▶). *Acta Cryst*. D**65**, 148–155]; the unit-cell characteristics take into account the presence of CHCl_3_. The dihedral angles between the planes of the naphthalene ring system and the meth­oxy­benzene rings are 71.05 (7) (*syn* to the central C=O group) and 57.27 (6)° (*anti* to the central C=O group). In the crystal, mol­ecules are linked by C—H⋯O inter­actions, generating *C*(12) chains running parallel to the *b* axis.

## Related literature   

For the uses and biological importance of naphthalene, see: Morikawa & Takahashi (2004 ▶); Rokade & Sayyed (2009 ▶).
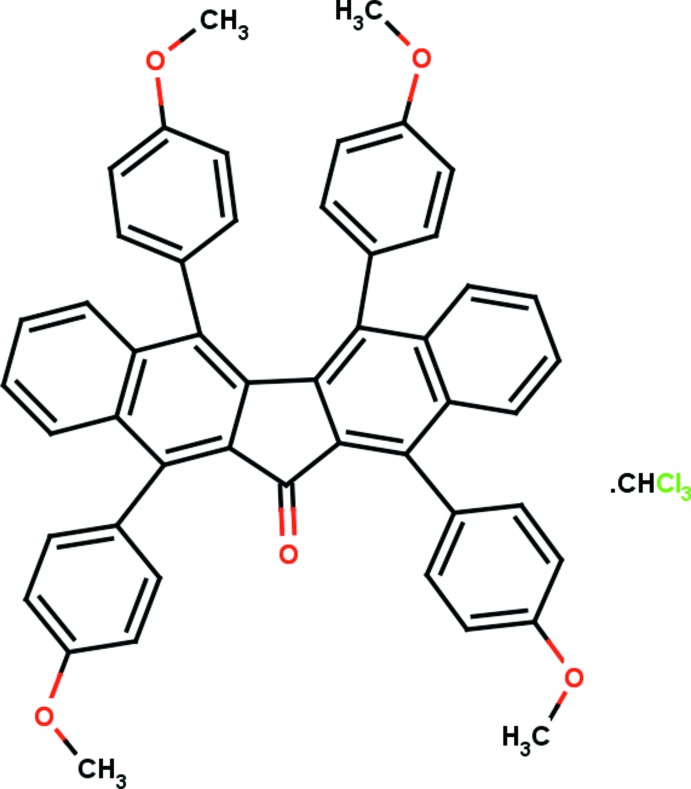


## Experimental   

### 

#### Crystal data  

C_49_H_36_O_5_·CHCl_3_
*M* *_r_* = 824.15
Monoclinic, 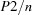

*a* = 9.7720 (4) Å
*b* = 12.0351 (4) Å
*c* = 17.3162 (6) Å
β = 93.947 (1)°
*V* = 2031.68 (13) Å^3^
*Z* = 2
Mo *K*α radiation
μ = 0.28 mm^−1^
*T* = 296 K
0.35 × 0.30 × 0.30 mm

#### Data collection  

Bruker Kappa APEXII CCD diffractometer
Absorption correction: multi-scan (*SADABS*; Bruker, 2008 ▶) *T*_min_ = 0.892, *T*_max_ = 0.934
20614 measured reflections
3474 independent reflections
2664 reflections with *I* > 2σ(*I*)
*R*_int_ = 0.025

#### Refinement  

*R*[*F* ^2^ > 2σ(*F* ^2^)] = 0.040
*wR*(*F* ^2^) = 0.125
*S* = 1.09
3474 reflections
248 parameters
H-atom parameters constrained
Δρ_max_ = 0.13 e Å^−3^
Δρ_min_ = −0.15 e Å^−3^

### 

Data collection: *APEX2* (Bruker, 2008 ▶); cell refinement: *APEX2*; data reduction: *SAINT* (Bruker, 2008 ▶); program(s) used to solve structure: *SHELXS97* (Sheldrick, 2008 ▶); program(s) used to refine structure: *SHELXL97* (Sheldrick, 2008 ▶); molecular graphics: *ORTEP-3 for Windows* (Farrugia, 2012 ▶) and *Mercury* (Macrae *et al.*, 2008 ▶); software used to prepare material for publication: *SHELXL97* and *PLATON* (Spek, 2009 ▶).

## Supplementary Material

Crystal structure: contains datablock(s) global, I. DOI: 10.1107/S1600536814016389/tk5322sup1.cif

Structure factors: contains datablock(s) I. DOI: 10.1107/S1600536814016389/tk5322Isup2.hkl

Supporting information file. DOI: 10.1107/S1600536814016389/tk5322Isup3.cml

CCDC reference: 1014030

Additional supporting information:  crystallographic information; 3D view; checkCIF report

## Figures and Tables

**Table 1:** Hydrogen-bond geometry (Å, °)

*D*—H⋯*A*	*D*—H	H⋯*A*	*D*⋯*A*	*D*—H⋯*A*
C24—H24c⋯O3^i^	0.96	2.39	3.199 (3)	141

Symmetry code: (i) 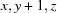.

**Fig. 1.**
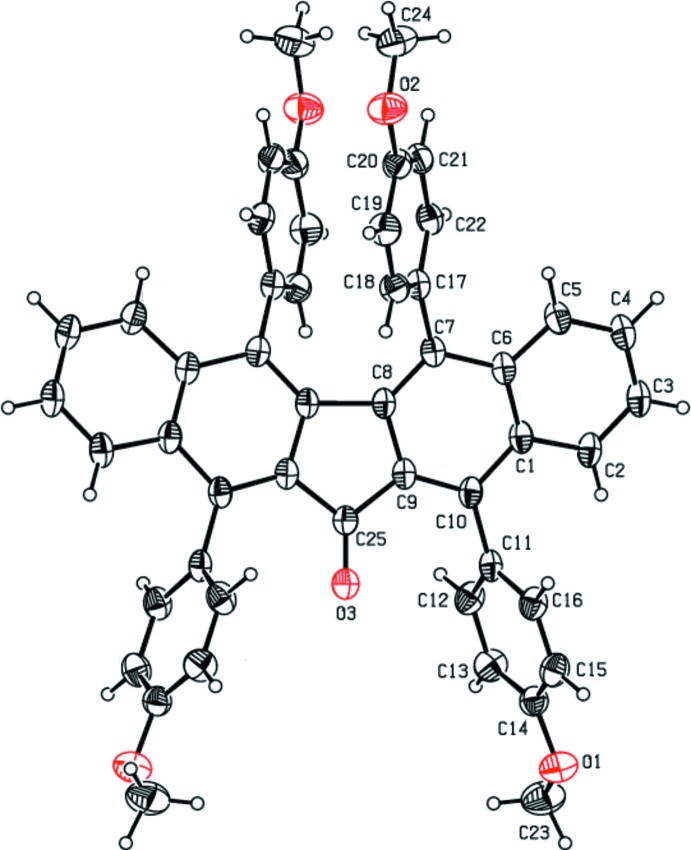
The molecular structure of the title compound (chloroform molecule omitted) showing the atom numbering scheme and displacement ellipsoids drawn at 30% probability level. The symmetry code of the unlabelled atoms is -x + 3/2, y, -z + 1/2.

**Fig. 2.**
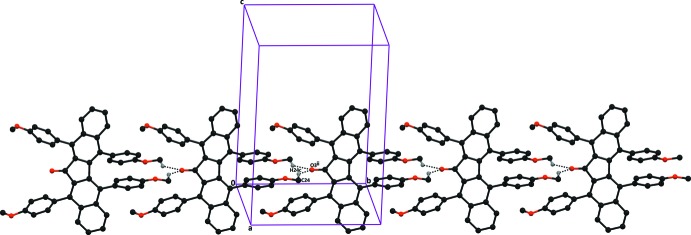
A partial packing packing diagram for the title compound viewed down the c axis, showing C24—H24c···O3ii hydrogen bonds resulting in the formation of C(12) chains running parallel to the b axis. [hydrogen atoms not involved in the hydrogen bonding and chloroform molecules have been omitted for the clarity]. Symmetry code: (ii) x, 1 + y, z.

## supplementary crystallographic information

### S2. Structural commentary

Naphthalene derivatives have been extensively employed in many fields and some posses important biological and commercial applications such as disinfe­cta­nts, insecticides, plant hormones and rooting agents (Morikawa & Takahashi, 2004). Naphthalene has been identified as new range of potent anti-microbiols effective against wide range of human pathogens (Rokade & Sayyed, 2009).

The title compound, C_49_H_36_O_6_·CHCl_3_, comprises half the organic molecule in the asymmetric unit, the complete molecule is being generated by two fold rotation, as well as half a disorderd chloro­form molecule. The X-ray analysis confirms the molecular structure and atom connectivity of the compound as illustrated in Fig. 1.

The dihedral angles between naphthalene ring and meth­oxy-substituted benzene rings are 71.05 (7) and 57.27 (6)°, respectively. The dihedral angle betweeen naphthalene and cyclo­penta-2,4-dienone residue is 13.26 (6)°.

In the crystal packing, molecules are linked *via* bifurcated C24—H24*c*···O3^ii^ inter­molecular hydrogen bonding, which generates *C*(12) infinite chains running parallel to the *b* axis. A view of the supra­molecular chain is shown in Fig. 2.

### S5. Synthesis and crystallization

To a solution of benzo[*c*]furan (0.20 g, 0.60 mmol) in dry di­chloro­methane (15 ml), indenone (0.24 g, 0.60 mmol) was added and refluxed till the disappearence of colour due to the benzo[*c*]furan (10 h). To this, PTSA (0.46 g, 2.42 mmol) was added (9 h). The reaction mixture was poured into saturated solution of NaHCO_3_ (40 ml), extracted with CHCl_3_ (3 x 15ml) and dried (Na_2_SO_4_). Removal of solvent followed by coloumn chromatographic purification (silica gel; 10% ethyl acetate in hexane) afforded the fluorenone derivative as a yellow solid. Single crystals suitable for X-ray diffraction were prepared by slow evapouration of a solution of the title compound in chloro­form at room temperature.

### S6. Refinement

The hydrogen atoms bound to the C atoms are treated as riding atoms, with d(C–H) = 0.93–0.96 Å, and with *U*_iso_(H) = 1.2–1.5*U*_eq_(C). The void in the crystal contains a highly disordered molecule of chloro­form which has beeen removed from the intensity data using the SQUEEZE routine in *PLATON* [Spek (2009). *Acta Cryst*. D65, 148–155]. The squeeze results show removal of 58 electrons equivalent of scattering materials each from two regions in the unit cell. This corresponds exactly to the number of electrons present per molecule of chloro­form.

### Crystal data

**Table:**

C_49_H_36_O_5_·CHCl_3_	*F*(000) = 740
*M**_r_* = 824.15	*D*_x_ = 1.347 Mg m^−^^3^
Monoclinic, *P*2/*n*	Mo *K*α radiation, λ = 0.71073 Å
Hall symbol: -P 2yac	Cell parameters from 3474 reflections
*a* = 9.7720 (4) Å	θ = 1.7–24.8°
*b* = 12.0351 (4) Å	µ = 0.28 mm^−^^1^
*c* = 17.3162 (6) Å	*T* = 296 K
β = 93.947 (1)°	Block, yellow
*V* = 2031.68 (13) Å^3^	0.35 × 0.30 × 0.30 mm
*Z* = 2	

### Data collection

**Table:**

Bruker Kappa APEXII CCD diffractometer	3474 independent reflections
Radiation source: fine-focus sealed tube	2664 reflections with *I* > 2σ(*I*)
Graphite monochromator	*R*_int_ = 0.025
ω &φ scans	θ_max_ = 24.8°, θ_min_ = 1.7°
Absorption correction: multi-scan (*SADABS*; Bruker, 2008)	*h* = −11→11
*T*_min_ = 0.892, *T*_max_ = 0.934	*k* = −14→14
20614 measured reflections	*l* = −20→20

### Refinement

**Table:**

Refinement on *F*^2^	Secondary atom site location: difference Fourier map
Least-squares matrix: full	Hydrogen site location: inferred from neighbouring sites
*R*[*F*^2^ > 2σ(*F*^2^)] = 0.040	H-atom parameters constrained
*wR*(*F*^2^) = 0.125	*w* = 1/[σ^2^(*F*_o_^2^) + (0.0692*P*)^2^ + 0.1579*P*] where *P* = (*F*_o_^2^ + 2*F*_c_^2^)/3
*S* = 1.09	(Δ/σ)_max_ < 0.001
3474 reflections	Δρ_max_ = 0.13 e Å^−^^3^
248 parameters	Δρ_min_ = −0.15 e Å^−^^3^
0 restraints	Extinction correction: *SHELXL97* (Sheldrick, 2008), Fc^*^=kFc[1+0.001xFc^2^λ^3^/sin(2θ)]^-1/4^
Primary atom site location: structure-invariant direct methods	Extinction coefficient: 0.0041 (10)

### Special details

**Table:**

Geometry. All e.s.d.'s (except the e.s.d. in the dihedral angle between two l.s. planes) are estimated using the full covariance matrix. The cell e.s.d.'s are taken into account individually in the estimation of e.s.d.'s in distances, angles and torsion angles; correlations between e.s.d.'s in cell parameters are only used when they are defined by crystal symmetry. An approximate (isotropic) treatment of cell e.s.d.'s is used for estimating e.s.d.'s involving l.s. planes.
Refinement. Refinement of *F*^2^ against ALL reflections. The weighted *R*-factor *wR* and goodness of fit *S* are based on *F*^2^, conventional *R*-factors *R* are based on *F*, with *F* set to zero for negative *F*^2^. The threshold expression of *F*^2^ > σ(*F*^2^) is used only for calculating *R*-factors(gt) *etc*. and is not relevant to the choice of reflections for refinement. *R*-factors based on *F*^2^ are statistically about twice as large as those based on *F*, and *R*- factors based on ALL data will be even larger.

### Fractional atomic coordinates and isotropic or equivalent isotropic displacement parameters (Å^2^)

**Table:**

	*x*	*y*	*z*	*U*_iso_*/*U*_eq_	
O1	0.70322 (14)	0.19072 (11)	−0.01039 (8)	0.0743 (4)	
O2	1.07295 (15)	1.23830 (11)	0.27627 (8)	0.0773 (4)	
O3	0.7500	0.48212 (15)	0.2500	0.0629 (5)	
C1	0.78181 (15)	0.71567 (14)	0.05173 (8)	0.0461 (4)	
C2	0.77909 (16)	0.69508 (16)	−0.02894 (8)	0.0524 (4)	
H2	0.7573	0.6243	−0.0476	0.063*	
C3	0.80786 (17)	0.77710 (16)	−0.07986 (9)	0.0575 (5)	
H3	0.8026	0.7626	−0.1327	0.069*	
C4	0.84506 (19)	0.88229 (16)	−0.05267 (9)	0.0600 (5)	
H4	0.8680	0.9371	−0.0873	0.072*	
C5	0.84809 (18)	0.90560 (16)	0.02447 (9)	0.0564 (4)	
H5	0.8746	0.9761	0.0416	0.068*	
C6	0.81187 (15)	0.82512 (14)	0.07917 (8)	0.0468 (4)	
C7	0.80505 (16)	0.85209 (13)	0.16025 (8)	0.0465 (4)	
C8	0.76176 (15)	0.76959 (13)	0.20778 (8)	0.0449 (4)	
C9	0.75067 (15)	0.65740 (13)	0.18137 (8)	0.0464 (4)	
C10	0.75817 (15)	0.62789 (13)	0.10526 (8)	0.0452 (4)	
C11	0.74288 (16)	0.51114 (14)	0.07819 (8)	0.0471 (4)	
C12	0.61738 (17)	0.45959 (15)	0.07359 (10)	0.0575 (5)	
H12	0.5419	0.4976	0.0903	0.069*	
C13	0.59946 (18)	0.35266 (15)	0.04487 (11)	0.0627 (5)	
H13	0.5132	0.3197	0.0424	0.075*	
C14	0.71000 (18)	0.29579 (14)	0.02007 (9)	0.0534 (4)	
C15	0.83762 (18)	0.34534 (16)	0.02556 (10)	0.0599 (5)	
H15	0.9134	0.3068	0.0098	0.072*	
C16	0.85313 (17)	0.45107 (15)	0.05413 (10)	0.0548 (4)	
H16	0.9398	0.4833	0.0575	0.066*	
C17	0.86294 (16)	0.95837 (13)	0.19126 (8)	0.0463 (4)	
C18	0.97613 (17)	0.95292 (14)	0.24523 (9)	0.0545 (4)	
H18	1.0070	0.8839	0.2631	0.065*	
C19	1.04222 (19)	1.04640 (15)	0.27230 (10)	0.0608 (5)	
H19	1.1177	1.0405	0.3078	0.073*	
C20	0.99703 (18)	1.15006 (14)	0.24698 (10)	0.0554 (4)	
C21	0.88557 (18)	1.15873 (15)	0.19489 (9)	0.0549 (4)	
H21	0.8545	1.2282	0.1780	0.066*	
C22	0.81949 (17)	1.06346 (14)	0.16761 (9)	0.0521 (4)	
H22	0.7437	1.0701	0.1324	0.063*	
C23	0.5727 (3)	0.14149 (19)	−0.02289 (16)	0.0952 (7)	
H23A	0.5322	0.1330	0.0257	0.143*	
H23B	0.5817	0.0699	−0.0465	0.143*	
H23C	0.5152	0.1880	−0.0564	0.143*	
C24	1.0313 (3)	1.34544 (17)	0.25541 (15)	0.0896 (7)	
H24A	1.0311	1.3536	0.2003	0.134*	
H24B	1.0935	1.3983	0.2801	0.134*	
H24C	0.9405	1.3583	0.2715	0.134*	
C25	0.7500	0.5833 (2)	0.2500	0.0468 (5)	

### Atomic displacement parameters (Å^2^)

**Table:**

	*U*^11^	*U*^22^	*U*^33^	*U*^12^	*U*^13^	*U*^23^
O1	0.0740 (9)	0.0585 (8)	0.0895 (10)	−0.0005 (7)	−0.0016 (7)	−0.0103 (7)
O2	0.0810 (10)	0.0622 (9)	0.0863 (10)	−0.0095 (7)	−0.0112 (7)	−0.0093 (7)
O3	0.0864 (13)	0.0581 (11)	0.0450 (9)	0.000	0.0103 (8)	0.000
C1	0.0364 (8)	0.0661 (11)	0.0362 (8)	−0.0031 (7)	0.0044 (6)	0.0009 (7)
C2	0.0466 (9)	0.0760 (11)	0.0348 (8)	−0.0049 (8)	0.0037 (6)	−0.0056 (8)
C3	0.0545 (10)	0.0845 (13)	0.0337 (8)	−0.0034 (9)	0.0052 (7)	0.0020 (8)
C4	0.0624 (11)	0.0786 (13)	0.0398 (9)	−0.0077 (9)	0.0087 (7)	0.0087 (9)
C5	0.0576 (10)	0.0685 (12)	0.0437 (9)	−0.0113 (8)	0.0072 (7)	0.0044 (8)
C6	0.0400 (8)	0.0644 (10)	0.0362 (8)	−0.0036 (7)	0.0045 (6)	0.0022 (7)
C7	0.0440 (9)	0.0581 (10)	0.0375 (8)	−0.0006 (7)	0.0041 (6)	0.0026 (7)
C8	0.0458 (9)	0.0565 (10)	0.0327 (8)	−0.0012 (7)	0.0045 (6)	0.0001 (7)
C9	0.0446 (9)	0.0598 (10)	0.0354 (8)	−0.0022 (7)	0.0066 (6)	0.0002 (7)
C10	0.0370 (8)	0.0613 (10)	0.0379 (8)	−0.0033 (7)	0.0073 (6)	−0.0039 (7)
C11	0.0462 (9)	0.0639 (10)	0.0314 (7)	−0.0036 (7)	0.0042 (6)	0.0003 (7)
C12	0.0416 (9)	0.0690 (12)	0.0627 (11)	−0.0015 (8)	0.0105 (7)	−0.0104 (9)
C13	0.0471 (10)	0.0662 (12)	0.0749 (12)	−0.0092 (8)	0.0048 (8)	−0.0065 (9)
C14	0.0564 (10)	0.0553 (10)	0.0480 (9)	0.0004 (8)	−0.0001 (7)	−0.0010 (8)
C15	0.0509 (10)	0.0713 (12)	0.0579 (10)	0.0069 (9)	0.0070 (8)	−0.0058 (9)
C16	0.0424 (9)	0.0687 (12)	0.0541 (10)	−0.0057 (8)	0.0080 (7)	−0.0070 (8)
C17	0.0456 (9)	0.0572 (10)	0.0365 (8)	−0.0026 (7)	0.0056 (6)	0.0022 (7)
C18	0.0574 (10)	0.0554 (10)	0.0498 (9)	0.0041 (8)	−0.0032 (7)	0.0042 (8)
C19	0.0582 (10)	0.0671 (12)	0.0549 (10)	−0.0007 (9)	−0.0119 (8)	0.0001 (8)
C20	0.0553 (10)	0.0579 (11)	0.0532 (10)	−0.0032 (8)	0.0053 (8)	−0.0036 (8)
C21	0.0607 (11)	0.0566 (10)	0.0483 (9)	0.0021 (8)	0.0094 (8)	0.0072 (8)
C22	0.0498 (9)	0.0648 (11)	0.0414 (8)	0.0004 (8)	0.0009 (7)	0.0076 (8)
C23	0.0904 (17)	0.0707 (14)	0.123 (2)	−0.0214 (12)	−0.0012 (14)	−0.0209 (13)
C24	0.1026 (18)	0.0595 (13)	0.1068 (18)	−0.0005 (12)	0.0071 (13)	−0.0168 (12)
C25	0.0465 (12)	0.0525 (15)	0.0421 (12)	0.000	0.0088 (9)	0.000

### Geometric parameters (Å, º)

**Table:**

O1—C14	1.370 (2)	C12—C13	1.386 (2)
O1—C23	1.410 (3)	C12—H12	0.9300
O2—C20	1.372 (2)	C13—C14	1.373 (2)
O2—C24	1.392 (2)	C13—H13	0.9300
O3—C25	1.217 (3)	C14—C15	1.380 (2)
C1—C2	1.417 (2)	C15—C16	1.370 (2)
C1—C6	1.424 (2)	C15—H15	0.9300
C1—C10	1.435 (2)	C16—H16	0.9300
C2—C3	1.366 (2)	C17—C22	1.387 (2)
C2—H2	0.9300	C17—C18	1.400 (2)
C3—C4	1.390 (3)	C18—C19	1.364 (2)
C3—H3	0.9300	C18—H18	0.9300
C4—C5	1.363 (2)	C19—C20	1.385 (2)
C4—H4	0.9300	C19—H19	0.9300
C5—C6	1.417 (2)	C20—C21	1.369 (2)
C5—H5	0.9300	C21—C22	1.383 (2)
C6—C7	1.447 (2)	C21—H21	0.9300
C7—C8	1.375 (2)	C22—H22	0.9300
C7—C17	1.484 (2)	C23—H23A	0.9600
C8—C9	1.427 (2)	C23—H23B	0.9600
C8—C8^i^	1.496 (3)	C23—H23C	0.9600
C9—C10	1.372 (2)	C24—H24A	0.9600
C9—C25	1.486 (2)	C24—H24B	0.9600
C10—C11	1.486 (2)	C24—H24C	0.9600
C11—C12	1.372 (2)	C25—C9^i^	1.486 (2)
C11—C16	1.385 (2)		
			
C14—O1—C23	117.79 (16)	O1—C14—C15	116.43 (16)
C20—O2—C24	118.67 (16)	C13—C14—C15	119.34 (16)
C2—C1—C6	118.69 (14)	C16—C15—C14	120.23 (16)
C2—C1—C10	121.01 (15)	C16—C15—H15	119.9
C6—C1—C10	120.29 (13)	C14—C15—H15	119.9
C3—C2—C1	121.29 (16)	C15—C16—C11	121.63 (16)
C3—C2—H2	119.4	C15—C16—H16	119.2
C1—C2—H2	119.4	C11—C16—H16	119.2
C2—C3—C4	120.01 (15)	C22—C17—C18	116.85 (15)
C2—C3—H3	120.0	C22—C17—C7	125.30 (14)
C4—C3—H3	120.0	C18—C17—C7	117.72 (14)
C5—C4—C3	120.45 (16)	C19—C18—C17	121.63 (16)
C5—C4—H4	119.8	C19—C18—H18	119.2
C3—C4—H4	119.8	C17—C18—H18	119.2
C4—C5—C6	121.66 (17)	C18—C19—C20	120.08 (15)
C4—C5—H5	119.2	C18—C19—H19	120.0
C6—C5—H5	119.2	C20—C19—H19	120.0
C5—C6—C1	117.69 (14)	C21—C20—O2	124.73 (16)
C5—C6—C7	121.72 (15)	C21—C20—C19	119.94 (16)
C1—C6—C7	120.59 (14)	O2—C20—C19	115.32 (15)
C8—C7—C6	117.10 (14)	C20—C21—C22	119.56 (16)
C8—C7—C17	122.09 (13)	C20—C21—H21	120.2
C6—C7—C17	120.16 (14)	C22—C21—H21	120.2
C7—C8—C9	120.74 (13)	C21—C22—C17	121.93 (15)
C7—C8—C8^i^	131.26 (10)	C21—C22—H22	119.0
C9—C8—C8^i^	107.44 (8)	C17—C22—H22	119.0
C10—C9—C8	123.01 (14)	O1—C23—H23A	109.5
C10—C9—C25	128.04 (15)	O1—C23—H23B	109.5
C8—C9—C25	108.43 (13)	H23A—C23—H23B	109.5
C9—C10—C1	116.85 (14)	O1—C23—H23C	109.5
C9—C10—C11	122.49 (14)	H23A—C23—H23C	109.5
C1—C10—C11	120.66 (13)	H23B—C23—H23C	109.5
C12—C11—C16	117.21 (16)	O2—C24—H24A	109.5
C12—C11—C10	121.07 (15)	O2—C24—H24B	109.5
C16—C11—C10	121.68 (14)	H24A—C24—H24B	109.5
C11—C12—C13	122.07 (16)	O2—C24—H24C	109.5
C11—C12—H12	119.0	H24A—C24—H24C	109.5
C13—C12—H12	119.0	H24B—C24—H24C	109.5
C14—C13—C12	119.50 (16)	O3—C25—C9	126.89 (10)
C14—C13—H13	120.2	O3—C25—C9^i^	126.89 (10)
C12—C13—H13	120.2	C9—C25—C9^i^	106.22 (19)
O1—C14—C13	124.23 (16)		
			
C6—C1—C2—C3	1.6 (2)	C1—C10—C11—C16	70.8 (2)
C10—C1—C2—C3	−176.88 (14)	C16—C11—C12—C13	−1.2 (3)
C1—C2—C3—C4	2.1 (3)	C10—C11—C12—C13	176.72 (16)
C2—C3—C4—C5	−2.5 (3)	C11—C12—C13—C14	0.0 (3)
C3—C4—C5—C6	−1.0 (3)	C23—O1—C14—C13	5.4 (3)
C4—C5—C6—C1	4.6 (2)	C23—O1—C14—C15	−174.72 (18)
C4—C5—C6—C7	−175.27 (16)	C12—C13—C14—O1	−178.90 (16)
C2—C1—C6—C5	−4.9 (2)	C12—C13—C14—C15	1.2 (3)
C10—C1—C6—C5	173.64 (14)	O1—C14—C15—C16	178.89 (16)
C2—C1—C6—C7	175.05 (14)	C13—C14—C15—C16	−1.2 (3)
C10—C1—C6—C7	−6.4 (2)	C14—C15—C16—C11	0.0 (3)
C5—C6—C7—C8	176.60 (15)	C12—C11—C16—C15	1.2 (2)
C1—C6—C7—C8	−3.3 (2)	C10—C11—C16—C15	−176.70 (15)
C5—C6—C7—C17	−12.5 (2)	C8—C7—C17—C22	−128.22 (17)
C1—C6—C7—C17	167.62 (13)	C6—C7—C17—C22	61.3 (2)
C6—C7—C8—C9	12.1 (2)	C8—C7—C17—C18	56.0 (2)
C17—C7—C8—C9	−158.68 (14)	C6—C7—C17—C18	−114.44 (17)
C6—C7—C8—C8^i^	−177.58 (19)	C22—C17—C18—C19	−1.2 (3)
C17—C7—C8—C8^i^	11.7 (3)	C7—C17—C18—C19	174.98 (16)
C7—C8—C9—C10	−11.7 (2)	C17—C18—C19—C20	0.6 (3)
C8^i^—C8—C9—C10	175.83 (15)	C24—O2—C20—C21	3.5 (3)
C7—C8—C9—C25	160.57 (13)	C24—O2—C20—C19	−177.98 (18)
C8^i^—C8—C9—C25	−11.85 (19)	C18—C19—C20—C21	0.2 (3)
C8—C9—C10—C1	1.6 (2)	C18—C19—C20—O2	−178.36 (16)
C25—C9—C10—C1	−169.11 (12)	O2—C20—C21—C22	178.03 (16)
C8—C9—C10—C11	−178.39 (14)	C19—C20—C21—C22	−0.4 (3)
C25—C9—C10—C11	10.9 (2)	C20—C21—C22—C17	−0.2 (3)
C2—C1—C10—C9	−174.37 (14)	C18—C17—C22—C21	1.0 (2)
C6—C1—C10—C9	7.2 (2)	C7—C17—C22—C21	−174.84 (15)
C2—C1—C10—C11	5.6 (2)	C10—C9—C25—O3	−3.49 (18)
C6—C1—C10—C11	−172.82 (13)	C8—C9—C25—O3	−175.31 (7)
C9—C10—C11—C12	73.0 (2)	C10—C9—C25—C9^i^	176.51 (18)
C1—C10—C11—C12	−107.00 (18)	C8—C9—C25—C9^i^	4.69 (7)
C9—C10—C11—C16	−109.20 (17)		

Symmetry code: (i) −*x*+3/2, *y*, −*z*+1/2.

### Hydrogen-bond geometry (Å, º)

**Table:**

*D*—H···*A*	*D*—H	H···*A*	*D*···*A*	*D*—H···*A*
C24—H24c···O3^ii^	0.96	2.39	3.199 (3)	141

Symmetry code: (ii) *x*, *y*+1, *z*.
